# MicroRNA-155 Mediates Augmented CD40 Expression in Bone Marrow Derived Plasmacytoid Dendritic Cells in Symptomatic Lupus-Prone NZB/W F1 Mice

**DOI:** 10.3390/ijms17081282

**Published:** 2016-08-06

**Authors:** Sheng Yan, Lok Yan Yim, Rachel Chun Yee Tam, Albert Chan, Liwei Lu, Chak Sing Lau, Vera Sau-Fong Chan

**Affiliations:** 1 Departments of Medicine, Li Ka Shing Faculty of Medicine, The University of Hong Kong, Hong Kong, China; ssyan@hku.hk (S.Y.); adayim@connect.hku.hk (L.Y.Y.); rach0806@connect.hku.hk (R.C.Y.T.); wkchanf@hku.hk (A.C.); 2 School of Public Health, Li Ka Shing Faculty of Medicine, The University of Hong Kong, Hong Kong, China; 3 Departments of Pathology, Li Ka Shing Faculty of Medicine, The University of Hong Kong, Hong Kong, China; liweilu@hkucc.hku.hk

**Keywords:** systemic lupus erythematosus, plasmacytoid dendritic cells, microRNAs, toll-like receptor 7

## Abstract

Systemic lupus erythematosus (SLE) is a chronic multi-organ autoimmune disease characterized by hyperactivated immune responses to self-antigens and persistent systemic inflammation. Previously, we reported abnormalities in circulating and bone marrow (BM)-derived plasmacytoid dendritic cells (pDCs) from SLE patients. Here, we aim to seek for potential regulators that mediate functional aberrations of pDCs in SLE. BM-derived pDCs from NZB/W F1 mice before and after the disease onset were compared for toll-like receptor (TLR) induced responses and microRNA profile changes. While pDCs derived from symptomatic mice were phenotypically comparable to pre-symptomatic ones, functionally they exhibited hypersensitivity to TLR7 but not TLR9 stimulation, as represented by the elevated upregulation of CD40, CD86 and MHC class II molecules upon R837 stimulation. Upregulated induction of miR-155 in symptomatic pDCs following TLR7 stimulation was observed. Transfection of miR-155 mimics in pre-symptomatic pDCs induced an augmented expression of *Cd40*, which is consistent with the increased CD40 expression in symptomatic pDCs. Overall, our results provide evidence for miR-155-mediated regulation in pDC functional abnormalities in SLE. Findings from this study contribute to a better understanding of SLE pathogenesis and ignite future interests in evaluating the molecular regulation in autoimmunity.

## 1. Introduction

Plasmacytoid dendritic cells (pDCs) constitute a unique subset of dendritic cells that have important immunoregulatory functions and play critical roles in autoimmunity. Similar to conventional or myeloid dendritic cells, pDCs upregulate the expressions of MHC class II antigen presentation molecules and T-cell costimulatory molecules such as CD80, CD86 and CD40 upon antigen stimulation, and serve as antigen presenting cells. Distinctively, pDCs specialize in producing type I interferon (IFN) [1]. pDCs express high levels of endosomal TLR7 and TLR9 which are the respective sensors for single stranded RNA and hypomethylated CpG DNA. Coupled with constitutive expression of the transcription factor interferon regulatory factor 7 (IRF7), pDCs are capable of rapidly secreting several hundred times more type I IFN than other leukocytes in response to viral or nucleic acids stimulations [2]. These unique features have granted pDCs a crucial role in the pathogenesis of systemic lupus erythematosus (SLE), a disease with multi-organ inflammatory manifestations mediated by autoantigens-autoantibodies immune complexes [3]. In human lupus disease, the role of pDCs is often inferred by the elevated serum level of IFN-α and upregulation of type I IFN-sensitive genes, which have been shown to correlate or associate with disease manifestations including the SLE hallmark anti-dsDNA autoantibodies, and the involvement of renal, hematological and central nervous systems [4,5,6]. Recent findings in animal studies, however, have specifically delineated pDCs’ functional contribution in SLE development. In the BXSB model, an early and transient depletion of pDCs before disease onset resulted in amelioration in lupus-associated pathology, which coincided with a reduced IFN-α/β-induced genes transcription [7]. Similar protective effects were also observed in *Tcf4*-haplodeficient *Tlr7* transgenic and B6*.Sle1.Sle3* lupus models in which the pDCs were functionally impaired by suppressing the E2-2 transcription factor [8].

In rheumatic diseases, a growing attention has been drawn to microRNAs (miRNAs) for their critical role in regulating immune cell functions [9,10]. These small non-coding RNA molecules post-transcriptionally regulate gene expression through complementary binding to their target messenger RNAs, leading to mRNA degradation or translation repression. Many regulatory miRNAs have been shown to link with SLE [11]. One interesting example is miR-146a. Its expression in peripheral blood mononuclear cells (PBMCs) of SLE patients negatively correlates with disease activity and IFN-sensitive genes expression [12]. Mechanistically, miR-146a acts as a negative regulator of type I IFN production by targeting multiple signaling components downstream of TLR7/9 and the retinoic acid-inducible gene-I pathways, thus its downregulated expression in lupus leukocytes promotes IFN-α production [12,13,14]. In addition, TLR7/9 and IFN-α stimulation of PBMCs induces miR-146a expression and apparently this negative feedback loop in IFN-α signaling pathway is dysfunctional in SLE patients [12]. Whether miR-146a-mediated IFN-α regulation is perturbed in lupus pDCs is unknown since there is no report on pDC-specific miRNA dysregulation in SLE so far. Nevertheless, a few recent studies have described the involvement of miR-155, miR-126, miR-29b, and miR-29c in regulating the functions of pDC in response to TLRs stimulation [15,16,17].

We previously demonstrated aberrations in frequencies, phenotypes and functions in DC subsets in SLE patients [18,19,20]. Intriguingly, bone marrow (BM)-derived pDCs from SLE patients were also found to have enhanced T-cell stimulatory ability and activated phenotypes [20], suggesting that abnormalities could originate from their precursors. Indeed, defects in BM and hematopoietic stem cells (HSCs) are also common in SLE patients [21,22,23]. Using the New Zealand Black/White F1 hybrid (NZB/W F1) lupus mouse model which mimics the spontaneous and multifactorial nature of human SLE disease, the present study aims to evaluate if SLE disease would impact on the generation and functional responses of BM-derived pDCs; and secondly to identify potential miRNA regulators in mediating functional irregularities in response to TLR stimulation.

## 2. Results

### 2.1. Lupus Disease Has Limited Impact on Plasmacytoid Dendritic Cells (pDC) Generation Potential of Bone Marrow (BM) Progenitor Cells in NZB/W F1 Mice

**Figure 1:**
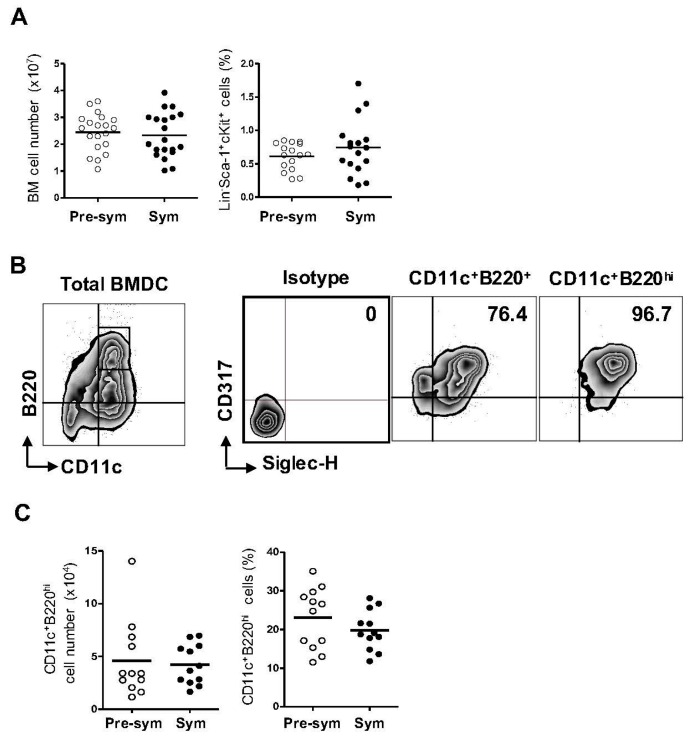
Gross development of pDCs is not affected by lupus. (**A**) Total numbers of bone marrow (BM) cells and frequencies of hematopoietic stem cells, marked by Lineage^−^ (Lin^−^) Sca-1^+^c-Kit^+^ (LSK), from pre-symptomatic (Pre-sym) and symptomatic (Sym) mice were compared. Collective data from more than 10 independent experiments, totaling *n* = 16–20 in the respective charts shown. Each symbol represents one mouse; (**B**) Cells generated from Flt3-ligand-supplemented BM culture at day-8 were analyzed. The expression of CD11c and B220 on live-gated cells as well as the expression of CD317 and Siglec-H on CD11c^+^B220^+^- or CD11c^+^B220^hi^-gated cells are shown in representative contour plots. Isotype antibodies control was used for the gating of CD317 and Siglec-H positive cells. Numbers shown are the percent of double positive cells; (**C**) Collective data from at least 3 independent experiments on the total numbers and frequencies of CD11c^+^B220^hi^ pDCs in the pre-symptomatic and symptomatic BM cultures are presented. Each symbol represents one mouse (*n* = 12).

To evaluate if the disease status in lupus has any effect on the development of pDCs, we isolated BM cells from the NZB/W F1 mice before (pre-symptomatic) and after (symptomatic) the onset of lupus symptoms for in vitro pDCs generation. In mice, the HSCs in BM are phenotypically identified as Lineage^−^ (Lin^−^), Sca-1^+^ and c-Kit^+^ (LSK) cells and from which the progenitors of pDCs arise [24]. We analyzed the total numbers of BM cells as well as the frequencies of LSK cells from both groups of mice (Figure 1A). Consistently, an average of (2.4 ± 0.7) × 10^7^ and (2.3 ± 0.8) × 10^7^ of total BM cells could be harvested from the pre-symptomatic and symptomatic mice respectively. The LSK frequencies in the total BM cells varied from 0.27% to 0.85% in the pre-symptomatic mice, and from 0.18% to 1.7% in the symptomatic mice. Overall, lupus disease did not have significant effect on the total number of BM cells or the LSK frequency in NZB/W F1 mice.

Cells derived from 8-day Flt3 ligand BM culture were evaluated for pDC generation. Mouse pDCs express the common DC marker CD11c and the B-cell lineage marker B220 [25]. Additionally, the BM stromal cell antigen 2 (BST2), also known as CD317 or PDCA-1, and the sialic acid-binding immunoglobulin (Ig)-like lectin H (Siglec-H) are specifically expressed on mouse pDCs at steady state [26,27]. Among these four markers, the former two can also be found on other immune cells while the expression of the latter two fluctuates upon stimulation. Therefore, a combination of CD11c, B220, CD317 and Siglec-H was used to identify pDCs from the heterogeneous BM culture (Figure 1B). The BM-derived DC culture constituted a mixture of cell populations expressing variable levels of these markers. Gating on the CD11c^+^B220^+^ cells, there remained a substantial proportion of cells that were CD317^−^ and/or Siglec-H^−^. In contrast, the CD11c^+^B220^hi^ subset were over 95% CD317^+^Siglec-H^+^, representing a more homogenous pDC population. Overall, approximately 20% of the cells generated from the Flt3 ligand-supplemented BM culture were CD11c^+^B220^hi^ pDCs. The frequencies as well as the total numbers of pDCs generated from the pre-symptomatic and symptomatic BM cells were comparable (Figure 1C).

### 2.2. BM-Derived pDCs Display Similar Phenotypes Irrespective of Lupus Disease Stage

**Figure 2:**
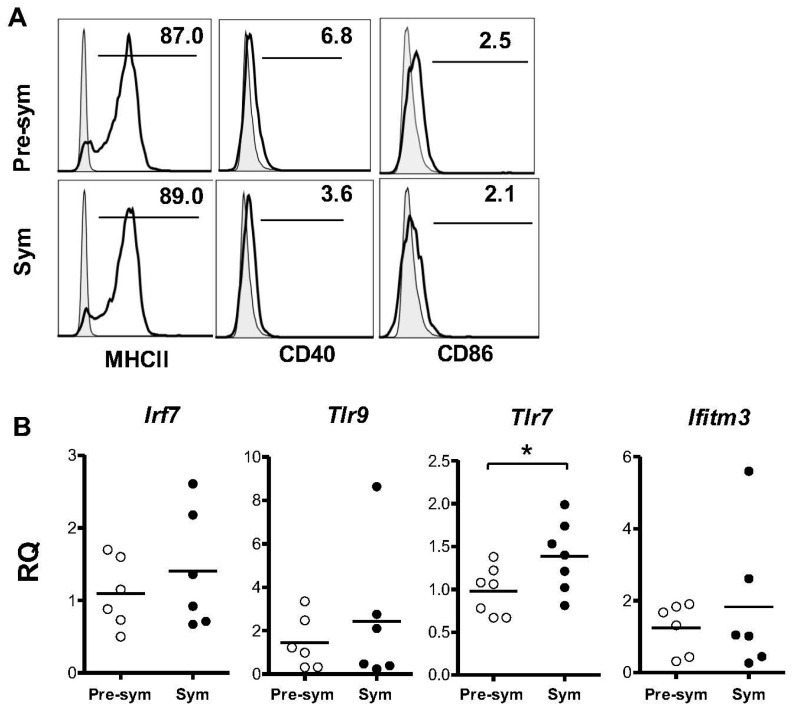

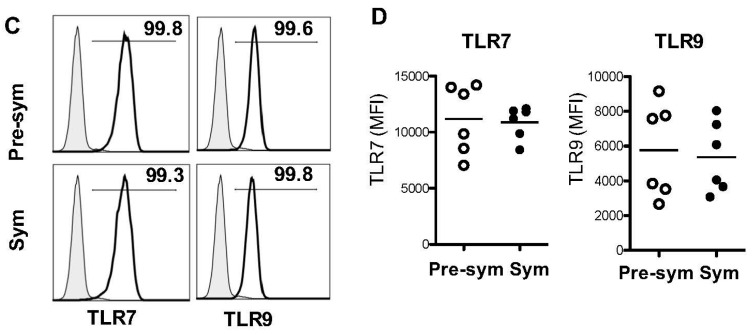
Bone marrow-derived pDCs from pre-symptomatic (Pre-sym) and symptomatic (Sym) lupus mice have similar phenotypes*.* (**A**) Expressions of MHC class II, CD40 and CD86 in CD11c^+^B220^hi^ pDCs derived from day-8 BM culture were analyzed. Representative plots of *n* = 6–7 is shown; (**B**) Expression of *Irf7*, *Tlr9*, *Tlr7* and *Ifitm3* of purified pDCs derived from day-8 BM culture were examined by real time PCR, and were shown as relative quantities (RQ) with reference to the average expression of corresponding genes in the pre-symptomatic pDCs. Collective data of *n* = 6–7 mice were shown; (**C**) Expressions of TLR7 and TLR9 in BM-derived pDCs on day-8 were analyzed. Numbers shown are percentages of positive cells. Shaded histograms represent isotype antibody staining controls; (**D**) Collective data comparing the mean fluorescence intensity (MFI) of TLR7 and 9 expressed in pre-symptomatic and symptomatic pDCs. Each symbol represents one individual mouse (*n* = 6). Data were collected from at least three independent experiments. *Bar:* mean values; * p ≤ 0.05 (unpaired two-tailed Student’s *t*-test).

Next, the surface expression of MHC class II and costimulatory molecules CD40 and CD86 were analyzed. Consistent with the reported pDC phenotypes at steady state [25,28], CD11c^+^B220^hi^ pDCs expressed moderate level of MHC class II and very low levels for CD40 and CD86 (Figure 2A), and similar expression levels of these markers were observed between pre-symptomatic and symptomatic mice. Using quantitative RT-PCR, the expressions of *Tlr7*, *Tlr9* and *Irf7* were also compared as they are constitutively expressed by pDCs and are essential to pDC functions. As shown in Figure 2B, no significant difference in *Tlr9* or *Irf7* expression was observed between the two groups. Interestingly, the pDCs derived from symptomatic mice displayed a small but significant increase in *Tlr7* expression when compared with the pre-symptomatic counterparts. However, further examination of TLR7 and TLR9 protein revealed high constitutive expression in these cells but no significant difference was observed between the two groups of pDCs (Figure 2C,D). In addition, the expression of three classical IFN-stimulated genes (ISGs) including IFN-induced protein with tetratricopeptide repeats 1 (*Ifit1*), IFN-inducible transmembrane protein 3 (*Ifitm3*) and 2′5′-oligoadenylate synthetase 1 (*Oas1*) were examined to check for any basal induction of type I IFN in lupus pDCs [29]. Among the three ISGs tested, no detectable expression was observed for *Ifit1* and *Oas1* (data not shown). Expression of *Ifitm3* was detectable but again there was no significant difference between the two groups of pDCs (Figure 2B). Seemingly, these results appear to contradict to the established role of type I IFN in SLE. A recent report has demonstrated in disease NZB/W F1 mice the elevated expression of IFN signature genes such as Mx1, Ifit2 and Cxcl10 in pDCs isolated from spleen and BM, thus supporting the involvement of type I IFN in this murine model [30]. Likely, these differentiated pDCs have been continuously exposed to and stimulated by TLR7/9-activating immune complexes in vivo. In contrast, in our system, BM pDC precursors were cultured in vitro with Flt3L, which is known to promote pDC differentiation but not activation. It is likely that these BM-derived pDCs did not spontaneously produce IFNα in culture and thus had limited basal or increase in the expression of the ISGs. Overall, these data suggested that in vitro pDC development from BM cells was not grossly affected by the development of lupus.

### 2.3. BM-Derived pDCs from Symptomatic Lupus Mice Show Heightened TLR7-Mediated Antigen Presentation and Costimulatory Molecules Expressions

**Figure 3:**
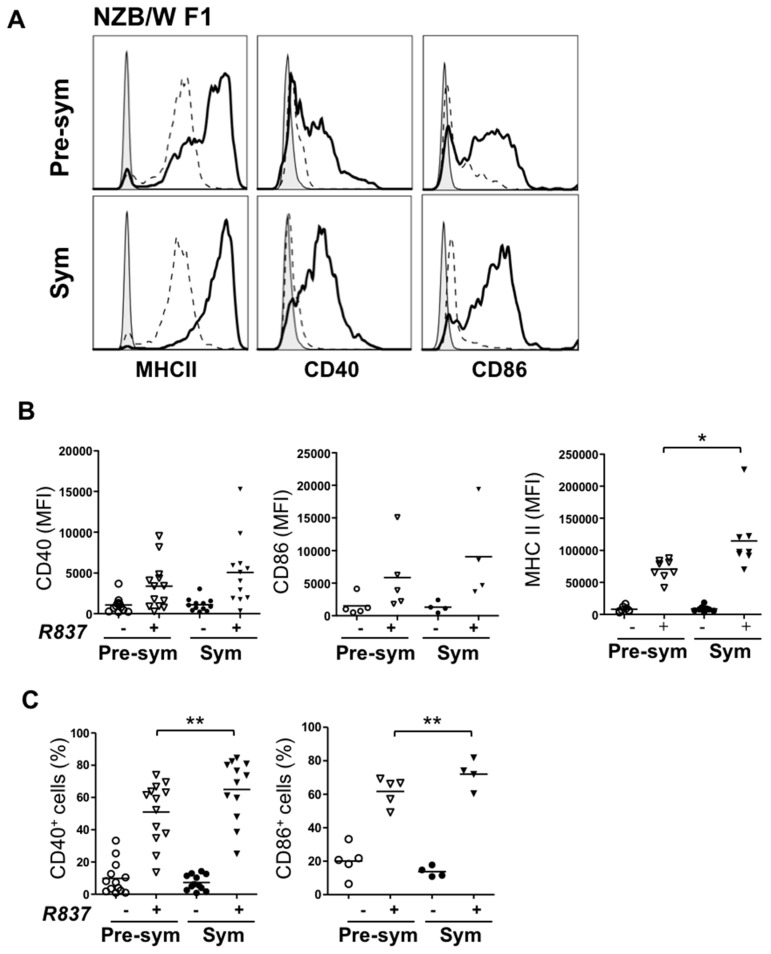
pDCs response to TLR7 stimulation is augmented in symptomatic mice. Purified BM-derived pDCs from pre-symptomatic (Pre-sym) or symptomatic (Sym) NZB/W F1 mice were treated with (+) or without (−) 5 µg/mL of R837 for 48 h. Expression of MHC class II, CD40 and CD86 were examined and shown as representative histograms in (**A**). *Grey*: Isotype; *dotted line*: unstimulated; *solid line*: R837 activated. Collective data illustrating changes in the mean fluorescence intensity (MFI) of MHC class II, CD40 and CD86 or percentages (%) of the latter two are shown in (**B**,**C**) respectively. Each symbol represents one experimental mouse. For expression of CD40, *n* = 12–13; CD86, *n* = 4–5; MHC class II, *n* = 8. Data were collected from at least three independent experiments. *Bar:* mean value. * *p* ≤ 0.05; ** p ≤ 0.01; (unpaired two-tailed Student’s *t*-test).

To examine the functional responses of pDCs, purified CD11c^+^B220^hi^ cells were activated by TLR agonists and compared. Upon R837 (TLR7 agonist) stimulation, MHC class II, CD40 as well as CD86 were clearly upregulated in both groups; and importantly, the induction was significantly higher in symptomatic mice (Figure 3). As depicted in Figure 3, pDCs from symptomatic group expressed augmented levels of MHC class II, and the percentage of cells expressing CD40 and CD86 was also significantly higher. In contrast to TLR7 response, no significant difference was observed in the induction of MHC class II, CD40 or CD86 expression upon TLR9 activation via CpG (Supplementary Figure S1). Additionally, TLR7/9-mediated IL-6 and IFN-α production was also compared. Similar amount of interleukin (IL)-6 was secreted by R837-activated pDCs from the two groups of mice (Supplementary Figure S2). Surprisingly, no detectable IFN-α was observed in the culture supernatant from R837-stimulated pDCs, whereas these pDCs were capable of producing IFN-α upon CpG stimulation. R837 is an imidazoquinoline amine analog to guanosine that commonly used for evaluation of TLR7 mediated IFN-α response in human cells and likely, R837 is inefficient in activating type I IFN production in mice [31,32].

**Figure 4:**
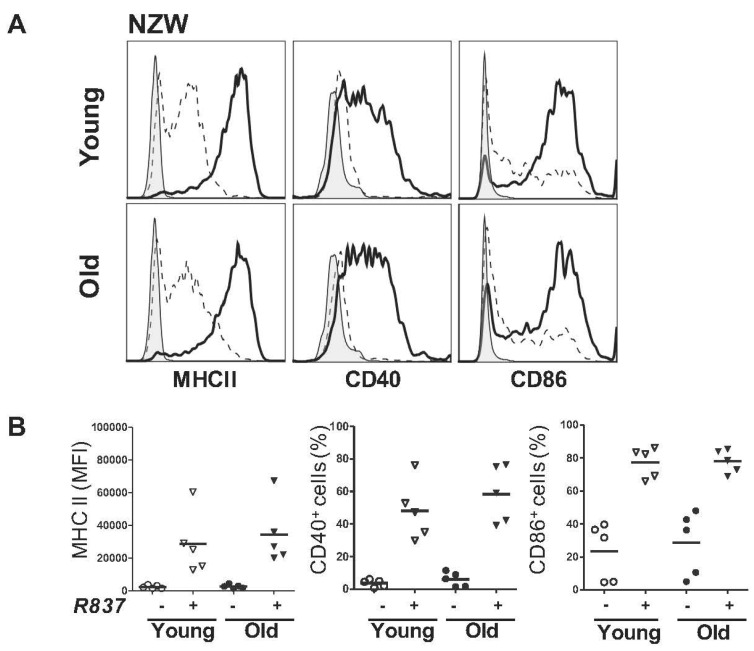
pDCs response to TLR7 stimulation is not affected in young and old NZW mice. Purified BM-derived pDCs from NZW mice age-matched with the pre-symptomatic (**A**, shown as Young) and symptomatic (**B**, shown as Old) NZB/W NZB/W F1 mice were treated with (+) or without (−) 5 µg/mL of R837 for 48 h. Expression of MHC class II, CD40 and CD86 were examined and shown as representative histograms in (**A**). *Grey*: Isotype; *dotted line*: unstimulated; *solid line*: R837 activated. Collective data illustrating changes in the mean fluorescence intensity (MFI) of MHC class II or percentages (%) of CD40 and CD86 are shown in (**B**). Each symbol represents one experimental mouse, *n* = 5. Data were collected from at least three independent experiments. *Bar:* mean value.

Apart from disease status, the age difference between the pre-symptomatic and symptomatic NZB/W F1 mice could have contributed to the differential TLR7 response. To evaluate this, R837 responses of pDCs derived from parental non-lupus NZW young (12–16 weeks old) and old (>36 weeks old) mice were compared (Figure 4). Upregulation of MHC class II, CD40 as well as CD86 on pDCs from both young and old NZW mice were induced and no significant differences were observed between the two groups. These results suggested that the elevated R837 response in symptomatic NZB/W F1 group was not likely attributed by the age factor.

### 2.4. TLR7 Mediated Overexpression of miR-155 in Lupus pDCs Contributes to the Heightened Cd40 Expression

**Figure 5:**
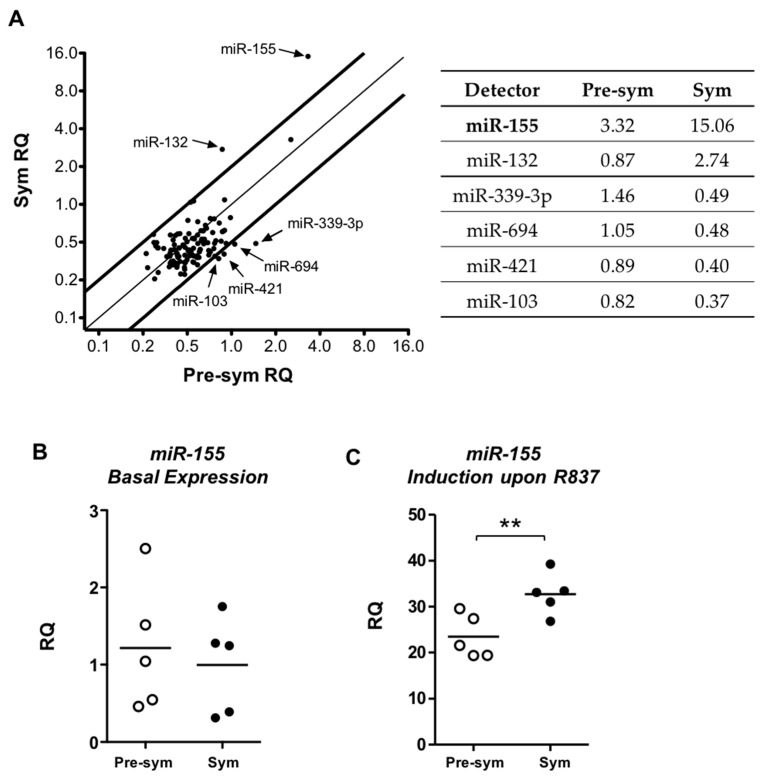
Induction of miR-155 is enhanced in symptomatic pDCs upon TLR7 activation. (**A**) Differential miRNAs expression profiles in R837-activated pDCs relative to unstimulated pDCs were analyzed. Relative quantities (RQ) of expressed miRNAs in symptomatic (Sym) group were plotted against pre-symptomatic (Pre-sym) group. The two bold lines indicate a cutoff of less than two-fold differences in expression. Arrows indicate miRNAs that are differentially expressed, whose mean RQ of three independent repeats are presented in the table. Expression of miR-155 was independently verified by real time PCR on separate samples of (**B**) unstimulated pDCs; and (**C**) R837-stimulated pDCs from pre-symptomatic and symptomatic mice. Each symbol represents sample from one experimental mouse. Data were collected from three independent experiments, *n* = 5 for each group of F1 mice. *Bar:* mean value. ** *p* ≤ 0.01 (unpaired two-tailed Student’s *t*-test).

To identify potential regulator(s) involved in the enhanced TLR7 response in lupus pDCs, we examined miRNA expression profiles upon R837 stimulation and compared between pre-symptomatic and symptomatic mice. The miRNA rodent set array included a total of 750 targets, among which 107 were expressed in pDCs. The basal expressions of miRNAs in unstimulated pDCs from symptomatic mice were first compared with the pre-symptomatic one and no consistent difference in specific miRNA was observed in three independent sets of profiling experiments (Supplementary Figure S3). Next, the relative quantity (RQ) of the expressed miRNAs in R837-activated pDCs was compared between the pre-symptomatic and symptomatic groups (Figure 5A). With a two-fold change as cutoff, six specific miRNAs were found differentially expressed. In particular, miR-155 was the most strongly and consistently upregulated upon activation, and with a significantly higher induction in the symptomatic group. A higher induction of miR-132 was also observed in symptomatic pDCs. The other four miRNAs, miR-339-3p, miR-694, miR-421 and miR-103 were downregulated in symptomatic group with only marginal changes in pre-symptomatic one. Since the differential induction of miRNAs was most pronounced in miR-155, we decided to focus on this miRNA by further validation with independent sets of stimulation samples using miR-155 specific qPCR assay. Consistent with the array data, miR-155 was expressed at similar levels in both groups of unstimulated pDCs (Figure 5B), while R837-induced miR-155 upregulation was significantly higher in the symptomatic group (23.44 ± 4.7 vs. 32.69 ± 4.5, Figure 5C). Furthermore, the R837-induced overexpression of miR-155 in pDCs of symptomatic NZB/W F1 mice was likely related to disease development since this difference was not observed when comparing young and old non-lupus NZW mice (Supplementary Figure S4).

**Figure 6:**
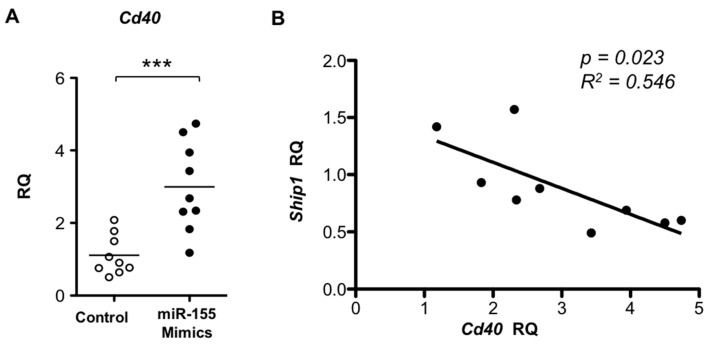
Transfection of miR-155 induces upregulation of *Cd40* in pre-symptomatic pDCs. (**A**) Pre-symptomatic pDCs were transfected with miR-155 mimics or scramble mimics control (control). The expression levels of *Cd40* in miR-155-transfected pDCs were examined by real time PCR and presented as relative quantity (RQ) to their respective mean expression in the control group. *Bar:* mean value. *** *p* ≤ 0.001 (unpaired two-tailed Student’s *t*-test); (**B**) Correlation of *Cd40* induction vs. *Ship1* suppression upon transfection of miR-155 mimics was plotted. The expression levels are presented as RQ relative to their respective average of controls in each group. The coefficient of correlation (R^2^) and *p* value were determined by linear regression. Each symbol represents one experimental mouse. Data were collected from at least three independent experiments (*n* = 9).

In order to study whether the elevated miR-155 expression contributes to pDC phenotypes observed in symptomatic mice, miR-155 mimics and scramble/non-targeting controls were transfected to pDCs derived from pre-symptomatic mice and *Cd40* expression was measured. A significant increase was observed for *Cd40* expression upon miR-155 overexpression (Figure 6A). This is consistent with the findings of an enhanced CD40 induction in symptomatic pDCs in response to R837 stimulation in association with a higher miR-155 induction (Figure 3 and Figure 5). In parallel, the expression of SH2-containing inositol phosphatase *Ship1*, a known primary target of miR-155 [33], was downregulated with a significant negative correlation with *Cd40* in the miR-155 overexpressing cells (Figure 6B).

## 3. Discussion

In this study, we have shown that in vitro derivation of pDCs from BM of symptomatic and pre-symptomatic NZB/W F1 mice was comparable phenotypically, including the expression of TLR7 and TLR9. However, hypersensitivity of symptomatic pDCs to TLR7 stimulation was illustrated by their increased upregulation of costimulatory molecules including CD40, CD86 as well as MHC class II. Screening of miRNAs in pDCs revealed an enhanced induction of miR-155 in symptomatic mice in response to TLR7 stimulation. Upon miR-155 overexpression, *Cd40* expression was significantly upregulated with a negative correlation to the miR-155 primary target *Ship1* expression. Overall, our findings suggest that the elevated miR-155 induction may contribute to the enhanced TLR7-induced CD40 expression in pDCs derived from BM of lupus mice.

In SLE patients, BM abnormalities are not rare and reported features include hypocellularity, necrotic alterations, reduced CD34^+^ HSC frequency as well as elevation of markers like CD95, CD123 and CD166 [21,22,23]. Such aberrations could be resulted from the disease itself and/or treatment using immunosuppressive drugs. Based on our previous findings in BM-derived pDCs from patients [20], we speculated that hypersensitivity in lupus pDCs could originate from abnormalities in BM precursors as a result of the disease. In NZB/W F1 mice, despite the lack of gross aberrations in BM cells and HSC frequency in symptomatic mice, BM-derived pDCs were indeed more sensitive to TLR7 stimulation and showed higher CD40, CD86 and MHC class II induction. These observations are consistent with the phenotypes observed in Flt3L-induced BM DCs from SLE patients [20]. Furthermore, myeloid DCs derived from BM of old disease NZB/W F1 females also showed differential response to estrogen modulation, leading to higher inflammatory cytokines production when compared with young pre-disease mice [34]. Thus, it is likely that the common DC progenitors in BM of lupus patients are modulated by disease parameters (e.g., perturbed cytokines levels) to give rise to hypersensitive DCs that exit to the periphery.

In lupus, activation of pDCs can be constantly triggered by self-nucleic acids in complex with autoantibodies or nuclear-binding proteins released from necrotic cells as well as activated neutrophils [35,36] via TLRs. The hyper-responsiveness to TLR7 stimulation in BM-derived pDCs in symptomatic lupus-prone mice likely represents one of the pathological attributes for SLE development. The pathogenic role of pDCs in lupus has been supported by recent evidence from selective depletion or functional blockade of this cell population in lupus-prone mouse models [7,8,37]. It was demonstrated that pDC deficiency in early disease development or the impairment of its function had beneficial effects including reduced splenomegaly, anti-nuclear autoantibody production, reduced glomerulonephritis and decreased levels of ISGs in kidney tissues [7,8]. In the tape-stripping model, depletion of pDCs protected NZB/W F1 mice from developing chronic skin lesions and so did the treatment of TLR7 and TLR9 inhibitors [37], suggesting that TLR7 and TLR9 signaling is important for lupus development. Indeed, the duplicated *Tlr7* gene, and hence the overexpressed receptor, in the BXSB male mice promoted a biased response of autoreactive B cells toward nuclear self-antigens [38]. Introducing the *Tlr7*-bearing *yaa* gene segment to B6.*Sle1* mice also accelerated autoimmunity, in which the disease severity positively correlated with the *Tlr7* expression level [39]. The pathogenic role of TLR9, however, is controversial. In fact, disparate contribution of TLR7 and TLR9 in SLE pathogenesis has been reported. In B6.*Nba2* congenic lupus mice, *Tlr9* deletion led to accelerated lupus with an increased production of anti-nuclear antibodies and augmented lupus nephritis, while disease progression in the *Tlr7/9* double-deficient mice was restored to a comparable or even slightly improved level as the parental strain [40]. Our finding is in agreement with the critical involvement of TLR7 signaling pathway in pDC malfunction in SLE pathogenesis while little implication can be derived from TLR9 response.

Despite having little baseline difference, the expression levels of CD40, CD86 and MHC class II molecules were further augmented in symptomatic BM-derived pDCs upon TLR7 stimulation. Consistently, splenic and lymph node pDCs in lupic NZB/W F1 mice have also been shown to display elevated expression of costimulatory molecules, including CD40 and CD86 in pDCs when compared with mice prior to disease onset [41,42], suggesting that the BM-derived pDCs bear similarities to the peripheral circulating pDCs. Similarly, a recent report by Zhou et al. has also demonstrated in disease NZB/W F1 mice a higher expression of MHC-II and CD80 in ex vivo splenic and BM pDCs when compared with pre-disease mice [30]. Likely, these peripheral pDCs in disease mice have been continuously exposed to and stimulated by TLR7 stimulating immune complexes in vivo*,* thus leading to the hyperactivated phenotypes upon ex vivo examination. Functionally, it is conceivable that a higher expression of CD86 and MHC class II could lead to better T cell stimulation by symptomatic pDCs, and indeed this has been observed in SLE patients [19,20]. Enhanced CD40 expression in pDCs may promote SLE development through its interaction with CD40L. Both soluble and cell-bound forms of CD40L have been shown to increase significantly in SLE patients [43,44]. Interestingly, it has been shown that activated platelets in lupus patient sera interact with pDCs through CD40L-CD40 signaling and potentiate type I IFN secretion by pDCs [45]. CD40 signaling can also synergize TLR9-induced type I IFN response in pDCs [46]. With the increased pDC hypersensitivity we found here and reported elsewhere, we believe that pDCs could potentially function as an accelerator to promote SLE development, at least in part through the increased CD40 expression upon TLR7 activation.

Previous miRNA profiling analyses in SLE were mainly done in total PBMCs from patients or total splenocytes and lymphocytes from murine models in unstimulated condition [12,47,48]. Here, changes in the miRNA expression were examined in purified pDCs upon TLR7 stimulation, and the heightened miR-155 induction was shown to mediate enhanced *Cd40* expression in symptomatic pDCs. This finding is in line with another study, which reported a decrease in MHC class II, CD40, and CD86 expressions in miR-155 knockdown Kupffer cells [49]. It is not clear how miR-155 mediates an increase in CD40 expression in pDCs. We observed a significant negative correlation between *Cd40* and *Ship1* expression in pDCs upon miR-155 modulation. SHIP1 is a negative immuno-regulator and has been shown to suppress TLR4 response via MyD88 in DCs [50]. Consistently, a recent report shows that miR-155 upregulation in DCs can lead to breaking of T cell tolerance by negative regulation of SHIP1 [51]. However, pertaining to this study, whether there is a functional correlation between CD40 and SHIP1 in pDCs is yet to be determined.

Our findings are consistent with the pro-autoimmune nature of miR-155, at least in the murine models. Notably, an upregulation of miR-155 was demonstrated in total splenocytes in different lupus mouse models, and was further shown in the splenic T and B lymphocyte compartments [48]. In MRL/lpr lupus mice, an enhanced miR-155 expression in CD4^+^CD25^+^Foxp3^+^ Treg cells was found in association with a compromised Treg suppressive function [52]; and the deletion of miR-155 in these mice alleviated lupus-like disease [53]. Besides, miR-155 appears to play a pathogenic role in other autoimmune diseases too. Silencing or depleting miR-155 in the mice protected them from developing experimental autoimmune encephalomyelitis or collagen-induced arthritis [54,55,56]. The miR-155-deficient mice in both of these models had impaired Th_17_ cell development and reduced proinflammatory Th_1_ and Th_17_ cytokines, suggesting a pathogenic contribution of miR-155 in T-cell mediated autoimmunity. Our study also suggests a pathogenic role of miR-155 in mediating pDC abnormality in the NZB/W F1 lupus model.

We identified a novel regulation of CD40 expression by miR-155, by which may contribute to the hyperactivated TLR7 response in lupus pDCs. Pertaining to our findings, more questions arise and warrant further exploration. First, it is not clear how the symptomatic BM-derived pDCs mediate higher TLR7 responses. We evaluated TLR7 as well as its downstream transcription factor *Irf7* expression levels and found no difference. Thus, other TLR7 downstream signaling networks (e.g., NF-κB and c-JNK pathways) should be examined. So far, the regulation of TLR7 (and TLR9) signaling network in pDCs remains elusive and can be influenced by other pDC-specific receptor-mediated signaling pathways [57]. A potential cross-regulation between TLR7 signaling and miR-155 would worth studying. In human pDCs, miR-155 and miR-155* are induced at different kinetics upon R837 stimulation [15]. Interestingly, miR-155 seems to act as a negatively regulator in type I IFN production by targeting TAB2 while miR-155* augments IFNα/β production by targeting IRAKM. In murine pDCs, R837 stimulation also upregulates miR-155 but cannot elicit IFNα production. We also found that miR-155 overexpression had minimal impact on *Tab2* expression (data not shown). In our miRNA screening assay, miR-155-3p (star form) was not included, and whether its expression changes upon TLR7 stimulation remains to be determined. It is likely that if a cross regulatory mechanism between murine TLR7 and miR-155 exists, it would be different from that found in human cells. In contrast to costimulatory molecules expression, it is not clear why the R837-induced IL-6 appears not be affected in symptomatic pDCs. For TLR9 stimulation, different CpG oligonucleotides elicit disparate functional outcomes depending on the nature of the phosphorothioated backbone as well as the presence of palindromic sequences. Class A CpG is an efficient type I IFN inducer while class B is more superior in inducing costimulatory molecules and cytokines expression in B cells, each triggering distinct regulatory pathways [46]. It is possible that different TLR7 agonists (e.g., ss-PolyU) may yield different response outcomes in our study system. More studies are therefore needed to evaluate if a different regulatory mechanism exists for TLR7-mediated cytokine modulation in lupus pDCs.

## 4. Experimental Section

### 4.1. Mice

The NZW/LacJ and NZB/BlNJ strains were purchased from The Jackson Laboratory (Bar Harbour, ME, USA). Breeding of the NZB/W F1 hybrid mice was carried out at The Laboratory Animal Unit, The University of Hong Kong. Licenses specifying experiments involved in this study were obtained from the Department of Health, Hong Kong. All experimental protocols and procedures were approved by the University Committee on the Use of Live Animals in Teaching and Research (CULATR 2213-10, approved on 5 August 2010 and CULATR 2449-11, approved on 25 May 2011). Lupus disease development in female NZB/W F1 mice and age-matched female NZW control mice was monitored. Serum levels of anti-dsDNA IgG autoantibodies were measured bi-weekly by ELISA. Proteinuria was measured weekly by Albutix stripes (Bayer HealthCare, Leverkusen, Germany). Aged (>25 weeks old) NZB/W F1 mice with persistent proteinuria (+++, 3 mg/mL or above for over 2 weeks) and anti-dsDNA IgG levels higher than two standard deviations (SD) over the mean of the age-matched NZW mice were considered as symptomatic. Young (8–15 weeks old) NZB/W F1 mice with anti-dsDNA levels comparable to non-lupus NZW control mice and negative for proteinuria were considered pre-symptomatic.

### 4.2. pDC Culture, Isolation and Stimulation

BM-derived pDCs were cultured as previously described with some modifications [24,28]. Briefly, total BM cells were harvested by flushing humeri, tibias and femurs of mice. Red blood cells were lysed using ACK buffer (0.15 M NH_4_Cl, 0.01 M KHCO_3_, 0.1 mM EDTA) and BM cells were seeded in 12-well plates at 5 × 10^6^ cells/mL in 10% fetal bovine serum in complete IMDM supplemented with 100 ng/mL of mouse Flt3 ligand (Peprotech, Rocky Hill, NJ, USA). In some experiments, hematopoietic stem cells were enriched before culture using the mouse lineage cell depletion kit (Miltenyi Biotec, Bergisch Gladbach, Germany). On day-8, cells were stained with appropriate antibodies and pDCs were purified by fluorescence-activated cell sorting using the Beckman Coulter MoFlo™ XDP cell sorter for subsequent experiments. Purified pDCs were seeded at 5 × 10^4^ cells/well in 96-well plates with 5 µg/mL of R837 (Imiquimod), 1 μM of CpG (InvivoGen, San Diego, CA, USA) or medium only (unstimulated) for 48 h, or specified otherwise. Stimulated cells were stained for activation markers and analyzed using the BD FACSCanto II Analyzer at Faculty Core Facilities, Li Ka Shing Faculty of Medicine, The University of Hong Kong. For staining of TLR7 and TLR9, day-8-BM cultures were first surface-stained for CD11c and B220, followed by fixation with 4% paraformaldehyde and permeabilization with 0.2% saponin. Intracellular staining was performed in the presence of 0.2% saponin throughout. The following anti-mouse monoclonal antibodies (BD Biosciences or Affymetrix eBiosciences, Franklin Lakes, NJ, USA) were used in this study: Sca-1-FITC (clone: D7); c-Kit-PE (2B8); CD11c-FITC (HL3); B220-APC or PECy7 (RA3-6B2); Siglec-H-PE (eBio440c); CD317-APC (eBio927); CD40-PE (3/23); CD86-PE or PECy7 (GL1); MHC class II—PECy7 (M5/114.15.2); TLR7-PE (A94B10) and TLR9-PE (J15A7).

### 4.3. Transfection of miRNA Mimics

Double-stranded miRNA mimics or scramble controls (GenePharma, Shanghai, China) were transfected into BM-derived pDCs using the Lipofectamine 2000^®^ reagent (Invitrogen, Thermo Fisher Scientific, Waltham, MA, USA) at 100 nM for 4 h at 37 °C. Total RNAs of the transfected cells were extracted using TRI reagent (Sigma-Aldrich, St. Louis, MO, USA) and stored at −80 °C for subsequent experiments.

### 4.4. Quantitative Real-Time RT-PCR

Quantitative gene expression analyses were performed following the Minimal Information for Publication of Quantitative Real-time PCR Experiments (MIQE) guidelines. Total cellular RNAs were extracted using TRI Reagent^®^ (Sigma-Aldrich, St. Louis, MO, USA) and then reverse transcribed into cDNAs with the ThermoScript™ RT-PCR system (Invitrogen, Thermo Fisher Scientific, Waltham, MA, USA) according to the manufacturer’s recommendation. Amplification of cDNAs was carried out using the StepOnePlus™ system and StepOne Software v2.3 (Applied Biosystems^®^, Thermo Fisher Scientific, Waltham, MA, USA) with KAPA SYBR^®^ Fast qPCR kit (Kapa Biosystems, Wilmington, MA, USA) following the thermal conditions at: 95 °C for 3 min and 40 cycles of 95 °C, 60 °C and 72 °C for 20 s each. Relative quantity (RQ) of gene-of-interest was normalized with β-actin and calculated using the formula: RQ = 2^−[ΔCq (test sample) − ΔCq (reference sample)]^. The primers for specific targets tested in this study are listed in Table 1. The amplification efficiency of each pair of primers was validated and was comparable to that of the endogenous control. In all experiments, no template control and melting curve analyses of the amplification products were included. Independent quantification of miR-155 was carried out using the miRNA assay (assay ID 002571 specific for mmu-miR-155) purchased from Applied Biosystems^®^ (Thermo Fisher Scientific, Waltham, MA, USA). Reactions and thermal conditions of quantitative real-time PCR for miR-155 were performed following manufacturer’s recommendation.

### 4.5. MicroRNA Profiling 

Extracted total RNAs were reverse transcribed using the Megaplex™ RT primers, Rodent pool A and B sets and the TaqMan^®^ miRNA reverse transcription kit. Subsequently, cDNAs were pre-amplified using the Megaplex™ PreAmp Primers and the TaqMan^®^ PreAmp Master Mix. Pre-amplified products were mixed with TaqMan^®^ Universal PCR master mix (No AmpErase^®^ UNG) in suggested ratio following the manufacturer’s recommendation and then loaded to the 384-well TaqMan^®^ Array Rodent miRNA A and B Cards. The reactions were performed on 7900HT Real-Time PCR System at The Centre for Genomic Sciences, The University of Hong Kong. Results were analyzed using Sequence Detection Systems v2.4 and RQ Manager V1.21. The reagents and analysis software used for the arrays were purchased from Applied Biosystems^®^ (Thermo Fisher Scientific, Waltham, MA, USA). The expression of each miRNA was normalized with the mammalian U6 internal control, and RQ was calculated using the formula: RQ = 2^−[ΔCq (test sample) − ΔCq (reference sample)]^.

### 4.6. Statistical Analysis

Unless specified, all experimental data were presented as mean values ± SD. Statistical significance was determined by unpaired two-tailed Student’s *t*-test, and correlation analysis by linear regression using GraphPad Prism (GraphPad, Inc., San Diego, CA, USA). 

## 5. Conclusions

Using the NZB/W F1 murine lupus model, we unraveled a miR-155-associated hyperactive TLR7 response in BM-derived pDCs after the onset of lupus. The expression of *Cd40* is upregulated upon overexpression of miR-155 in pDCs. In essence, the current study highlights the elevated pDC responses to TLR7 stimulation in lupus and suggests a potentially pathogenic role mediated by miR-155. We present here the connection between miRNA and the hyperactivation of pDCs including the upregulation of CD40. Findings in this study shed light on the potential roles of pDCs in the pathogenesis of SLE.

## Figures and Tables

**Table 1:** List of primers used in this study.

Gene	Sequence		Accession No.	Tm	Pdt	Location
*β-actin*	Fwd	TTG CTG ACA GGA TGC AGA AG	NM_007392.3	58.2	147	1039–1058
	Rev	TGA TCC ACA TCT GCT GGA AG	–	57.3	–	1185–1166
*Irf7*	Fwd	GAT CTT CAA GGC CTG GGC TGT GG	NM_016850.3	57.1	220	611–633
	Rev	TCC AAG CTC CCG GCT AAG TT	–	55.0	–	830–811
*Ifitm3*	Fwd	GAT CGG CTT CTG TCA GAA CTA	NM_025378.2	57.2	154	209–229
	Rev	TTC CGA TCC CTA GAC TTC ACG GA	–	62.5	–	362–340
*Tlr7*	Fwd	TGT TAC TAT TCC ATA CCT GGC CAC	NM_133211.4	60.1	179	2640–2663
	Rev	GGT GAC TTG TTG TCA TAA CTA CC	–	57.1	–	2818–2796
*Tlr9*	Fwd	CAA CAT GGT TCT CCG TCG AA	NM_031178.2	58.2	243	103–122
	Rev	TTG TGC AGG TGG TGG ATA CGG T	–	64.2	–	345–324
*Cd40*	Fwd	TTG TTG ACA GCG GTC CAT CT	NM_011611.2	59.6	154	112–131
	Rev	CTG AGT CAC ATG GGT GGC AT	–	60.0	–	265–246
*Ship1*	Fwd	CCA GGG CAA GAT GAG GGA GA	NM_001110193.2	60.98	195	2766–2785
	Rev	GGA CCT CGG TTG GCA ATG TA	–	60.04	–	2960–2941

Fwd: forward primer; Rev: reverse primer; Tm: melting temperature; Pdt: size of the amplification product in base pair. Final concentration of primer used in qPCR was 200 nM.

## Supplementary Materials

The following are available online at www.mdpi.com/1422-0067/17/8/1282/s1.
