# Supplementary material for: MicroRNA-155 Mediates Augmented CD40 Expression in Bone Marrow Derived Plasmacytoid Dendritic Cells in Symptomatic Lupus-Prone NZB/W F1 Mice
Source: Int J Mol Sci. 2016 Aug 6;17(8):1282. doi: 10.3390/ijms17081282 (PMC5000679; doi:10.3390/ijms17081282)
Supplement: Supplementary file 1 [file ijms-17-01282-s001.pdf]

Supplementary Materials: MicroRNA-155 Mediates Augmented CD40 Expression in Bone Marrow Derived Plasmacytoid Dendritic Cells in Symptomatic Lupus-Prone NZB/W F1 Mice

Sheng Yan, Lok Yan Yim, Rachel Chun Yee Tam, Albert Chan, Liwei Lu, Chak Sing Lau and Vera Sau-Fong Chan

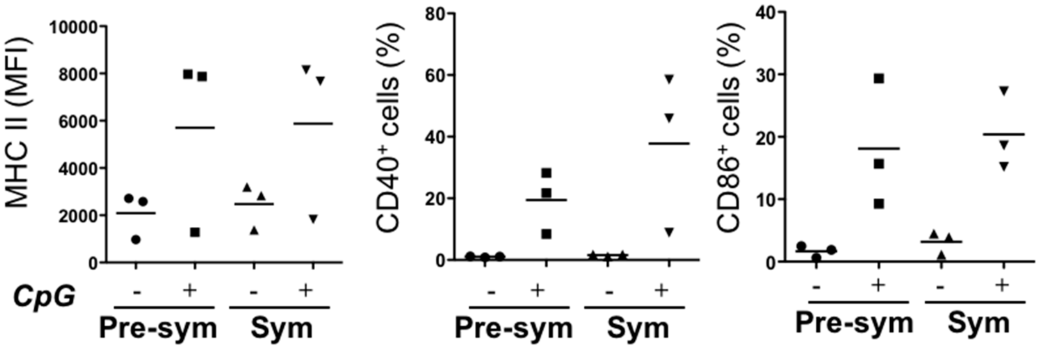

Figure S1. pDCs response to TLR-9 stimulation is not affected in SLE. Purified BM-derived pDCs from pre-symptomatic (Pre-sym) or symptomatic (Sym) NZB/W F1 mice were treated with (+) or without (−) 1 μM of CpG for 48 h and examined for expression of MHC class II, CD40 and CD86. Collective data illustrating changes in the mean fluorescence intensity (MFI) of MHC class II or percentages (%) of CD40 and CD86 are shown. Each symbol represents one experimental mouse ($n = 3$). *Bar:* mean value. No statistical significances can be found (two-tailed Student's *t*-test, unpaired).

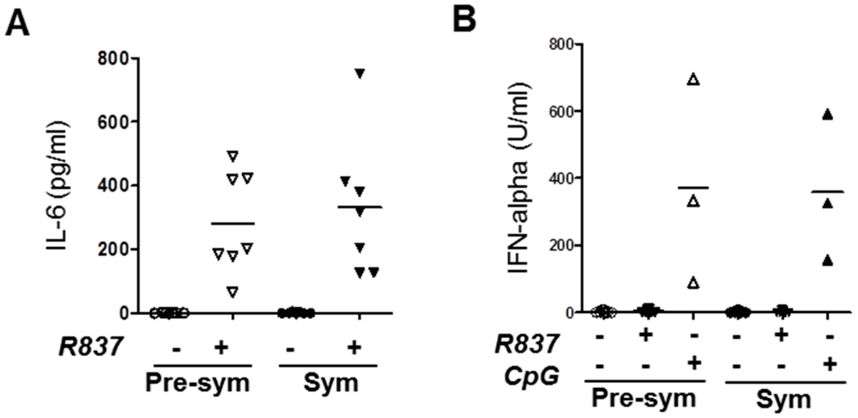

Figure S2. Production of IFN-*α* or IL-6 by activated pDCs is not affected by lupus. Secretion of (**A**) IL-6 and (**B**) IFN-*α* by BM-derived pDCs from pre-symptomatic (Pre-sym) and symptomatic (Sym) NZB/W F1 mice was analyzed following incubation with (+) or without (-) R837 (5 μg/mL) or CpG (1 μM) for 48 h by ELISA. Each symbol represents one experimental mouse. For IL-6, $n = 7$; for IFN-α with R837 stimulation, $n = 9$; for IFN-α with CpG stimulation, $n = 3$. *Bar:* mean value. No statistical significances can be found (two-tailed Student's *t*-test, unpaired).

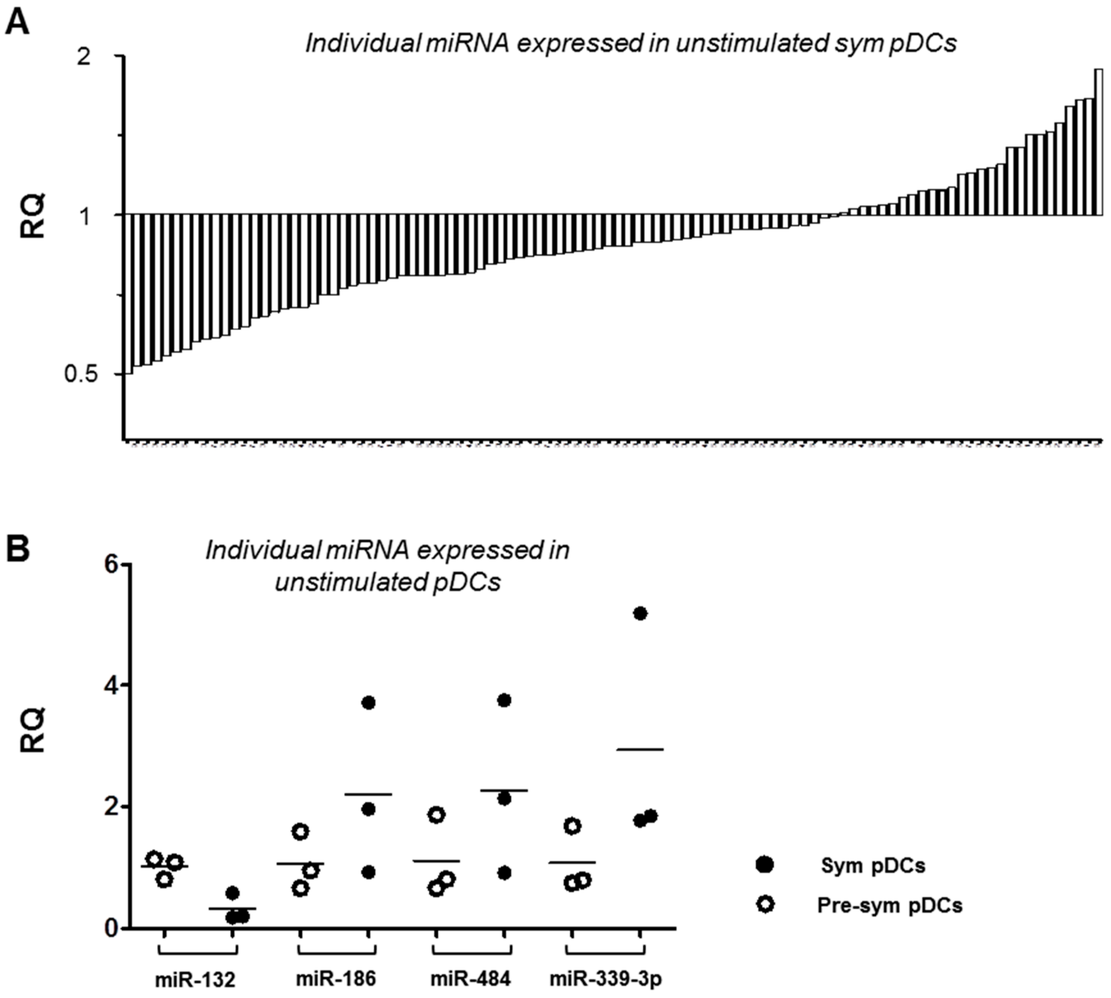

Figure S3. miRNA expression profile in unstimulated BM-derived-pDCs is comparable between pre-symptomatic and symptomatic mice. (**A**) The miRNA expression profiles in unstimulated pDCs from symptomatic (Sym) NZB/W F1 mice were compared with pre-symptomatic (Pre-sym) pDCs and presented as relative quantities (RQ). Each column represents the average expression of an individual miRNA target from three independent experiments. Majority of the miRNAs had a RQ within the two-fold cut off except 4 miRNAs that were further analyzed individually and results were illustrated in (**B**). The miRNAs with lowest (miR-132) and top three highest expressions (miR-186, miR-484 and miR339-3p) in unstimulated sym pDCs were compared with pre-sym pDCs. Each symbol represents one experimental mouse. *Bar:* mean values. No statistical significance can be found (two-tailed Student's *t*-test, unpaired).

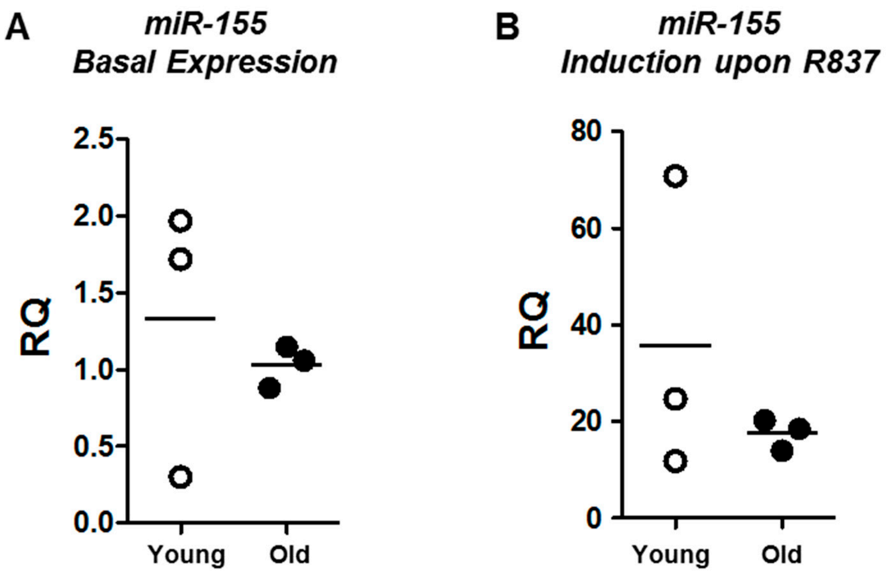

Figure S4. Induction of miR-155 upon TLR7 stimulation is comparable in young and old NZW mice. Expression of miR-155 in unstimulated pDCs (**A**) and R837-stimulated pDCs (**B**) from young (Young, open circles) and old NZW mice (Old, black circles) were compared. Each symbol represents sample from one experimental mouse (n = 3). *Bar:* mean value. N statistical significance can be found (two-tailed Student's t-test, unpaired).
